# The Effect of Compassion Satisfaction on Happiness at Work Among Nurses: Its Role as Mediator of Employee Well-Being

**DOI:** 10.3390/bs16091676

**Published:** 2026-09-17

**Authors:** Halil Türktemiz

**Affiliations:** Department of Health Management, Faculty of Health Sciences, Sakarya University of Applied Sciences, Sakarya 54400, Türkiye; halilturktemiz@subu.edu.tr

**Keywords:** compassion satisfaction, happiness at work, employee well-being, nursing

## Abstract

Nurses play a significant role in the delivery of healthcare services. They experience compassion satisfaction when providing care to patients in need. However, the effect of compassion satisfaction on workplace happiness has not been sufficiently examined. Drawing on self-determination theory and positive psychology, this study examines the mediating role of employee well-being in the relationship between compassion satisfaction and workplace happiness among nurses. The findings indicate that nurses’ compassion satisfaction has a statistically significant positive effect on workplace happiness and employee well-being. Employee well-being fully mediates the relationship between compassion satisfaction and workplace happiness. This finding suggests that nurses’ compassion satisfaction alone is insufficient to directly increase workplace happiness; however, improving employee well-being can increase nurses’ workplace happiness. It is recommended that staffing levels be adjusted based on nurses’ workloads, that managers adopt a nurse-centered leadership style, and that flexible work hours be integrated into the system. These measures can lead to greater happiness among nurses and improved quality of care.

## 1. Introduction

By administering treatments to patients in accordance with physicians’ treatment plans, nurses play a vital role in the efficient delivery of healthcare services. Nurses provide their patients with compassionate and empathetic care and treatment. Compassion is defined as the ability to perceive another person’s distress and the willingness to alleviate it (Desai et al., 2024). Nurses typically experience compassion satisfaction (CS) when they contribute to the recovery of patients affected by sudden, life-threatening illnesses or events that cause permanent damage and provide comfort to these patients (Sacco et al., 2015). The literature distinguishes between two concepts: “compassion fatigue” and “CS.” While compassion fatigue refers to negative states, such as burnout, that result from prolonged work, CS refers to the positive sense of fulfillment that nurses derive from work performed for the benefit of patients (Mansur et al., 2022). The positive emotion that arises from nurses’ awareness of helping patients is referred to as CS (Durkin et al., 2016). When CS predominates, nurses are more likely to experience professional satisfaction, demonstrate greater resilience, and support safe patient care (Patil et al., 2025). Since nurses derive satisfaction from understanding patients’ perspectives, providing emotional support, and meeting their needs, the fulfillment derived from compassion is a desirable outcome in nurse–patient interactions (Nadarajan et al., 2025). Compassion is essential not only for patient care and nurses’ well-being but also for raising standards within the healthcare system (Papadopoulos et al., 2016).

Happiness is the degree to which an individual positively evaluates the overall quality of their own life (Veenhoven, 2021). Happiness can be defined as the experience of positive emotions and the pleasure derived from them. Happiness is a subjective concept shaped by individuals’ living conditions, needs, and expectations (Uzunbacak et al., 2019). Happiness at work (HAW) can protect individuals during crises by reducing the harmful effects of negative emotions on concentration, creativity, and related aspects of functioning (Kim et al., 2021). Happiness and inner satisfaction with life can serve as intrinsic motivators for nurses to perform their duties (Chang et al., 2020). Since nurses play a critical role in delivering healthcare services, they need to experience a sense of well-being to provide effective patient care; therefore, promoting HAW among nurses is essential. HAW is shaped not only by the work performed but also by individuals’ subjective feelings (Güner & Bozkurt, 2017). High levels of CS can be associated with factors such as high levels of happiness, optimism, life satisfaction, and social connection (Heffernan et al., 2010; Mantelou & Karakasidou, 2019). HAW also enables nurses to perform their duties more comfortably and to work more efficiently to achieve organizational goals. Subjective happiness is a significant factor that enables nurses, who play a crucial role in healthcare services, to continue working while maintaining their health (Takeda et al., 2020). Nurses’ happiness at work is sustained by their relationships with patients (Taş, 2023). Therefore, nurses’ CS may support their happiness.

Well-being may generally be understood as a state of wellness (Yılık & Yüksel, 2023). Well-being reflects a person’s positive feelings toward the world (Özkan & Gürbüz, 2019). Employee well-being (EWB) encompasses all aspects of working life, ranging from the safety of the physical environment to the workplace atmosphere and the organization of work (Yaşar & Özfırat, 2024). EWB is related not only to environmental factors in the workplace but also to relationships with coworkers. Because nurses interact with patients, their families, and the community, ensuring nurses’ EWB has direct implications for the delivery of healthcare services and social policy (Xiao et al., 2022). Supporting professional development to enhance nurses’ skills and knowledge, along with initiatives to develop managers’ competencies, can contribute to increased EWB (O’Connor et al., 2024). In addition, incorporating self-care elements such as breathing exercises, maintaining a sleep schedule, practicing gratitude, and expressing emotions into nursing routines can support EWB and improve patient care (Patil et al., 2025). Appropriate workplace conditions are expected to facilitate more efficient work processes, reduce stress levels, and foster a happy workforce. Initiatives aimed at supporting nurses’ well-being can help them remain happy when facing work-related challenges and foster positive states such as optimism (Aljohani et al., 2025). Nurses’ well-being should not be considered solely from an individual perspective. It should be viewed as a component of nursing care quality and organizational performance. It has been suggested that nursing services may influence factors such as clinical complexity and length of hospital stay in pediatric patients (Cesare et al., 2024). Similarly, employee well-being may serve as a link between positive professional experiences, such as nurses’ compassion satisfaction, and job satisfaction.

This study is grounded in positive psychology and draws on the central tenets of self-determination theory (SDT). Positive psychology focuses on positive experiences and their manifestations, including well-being, happiness, personal strengths, wisdom, creativity, imagination, and the characteristics of positive groups and organizations (Hefferon & Boniwell, 2011). Seligman and Csikszentmihalyi (2000), two pioneers of positive psychology, similarly state that positive psychology examines positive experiences and their manifestations by focusing on well-being, happiness, personal strengths, wisdom, creativity, imagination, and the characteristics of positive groups and institutions within the broader field of psychology. According to SDT, satisfying individuals’ psychological needs for autonomy, competence, and relatedness increases intrinsic motivation and psychological well-being. From this perspective, nurses’ CS is conceptualized as an indicator of the fulfillment of their needs for competence and meaning in a professional context. SDT further suggests that satisfying such needs promotes employee well-being, which, in turn, contributes to workplace happiness. Based on this theoretical framework, the following hypotheses were developed:

This study focuses on CS, one of the positive dimensions experienced by nurses while practicing their profession, within the framework of positive psychology theory (Seligman & Csikszentmihalyi, 2000). Within the framework of self-determination theory, it is argued that CS enhances nurses’ HAW by supporting not only the fulfilment of their relational needs but also their needs for competence and autonomy (Ryan & Deci, 2000). Based on this evidence, Hypothesis 1 was formulated as follows:

**H_1_.**  *Nurses’ compassion satisfaction has a significant effect on their happiness at work*.

From the perspective of SDT, which considers the perceived forces that guide individuals, EWB arises from the fulfilment of the needs for relatedness, competence and autonomy (Deci & Ryan, 2000). In this context, it is suggested that nurses’ CS increases EWB by fulfilling their need for relationality through the bond established in the patient treatment process, their need for competence through positive outcomes in patient care, and their need for autonomy through the intrinsic CS (Ryan & Deci, 2000). Based on this theoretical rationale, Hypothesis 2 was formulated as follows:

**H_2_.**  *Nurses’ compassion satisfaction has a significant effect on their employee well-being*.

According to the theory of positive psychology, it is a developmental process that focuses not only on the absence of negative emotions but also on one’s strengths and positive experiences (Seligman & Csikszentmihalyi, 2000). On the other hand, within the framework of SDT, meeting an individual’s needs for competence, autonomy and relatedness directly contributes to their well-being (Deci & Ryan, 2000). Accordingly, nurses whose physical, mental and emotional needs are met are expected to be HAW as a reflection of their psychological well-being. Based on this information, the H_3_ hypothesis was formulated as follows:

**H_3_.**  *Nurses’ employee well-being has a significant effect on their happiness at work*.

Based on positive psychology, an individual’s functioning is viewed not merely as the absence of negativity, but also as the development of positive experiences (Seligman & Csikszentmihalyi, 2000). According to SDT, the CS nurses derive from patient care enhances their happiness. Furthermore, according to SDT, meeting the fundamental needs for autonomy, competence and relatedness in the workplace fosters EWB and supports nurses’ happiness (Deci & Ryan, 2000). Based on this information, hypothesis H4 was formulated to examine the mediating role of EWB in the effect of CS on HAW.

**H_4_.**  *Employee well-being mediates the effect of nurses’ compassion satisfaction on their happiness at work*.

## 2. Materials and Methods

### 2.1. Study Design and Setting

**Figure 1:**
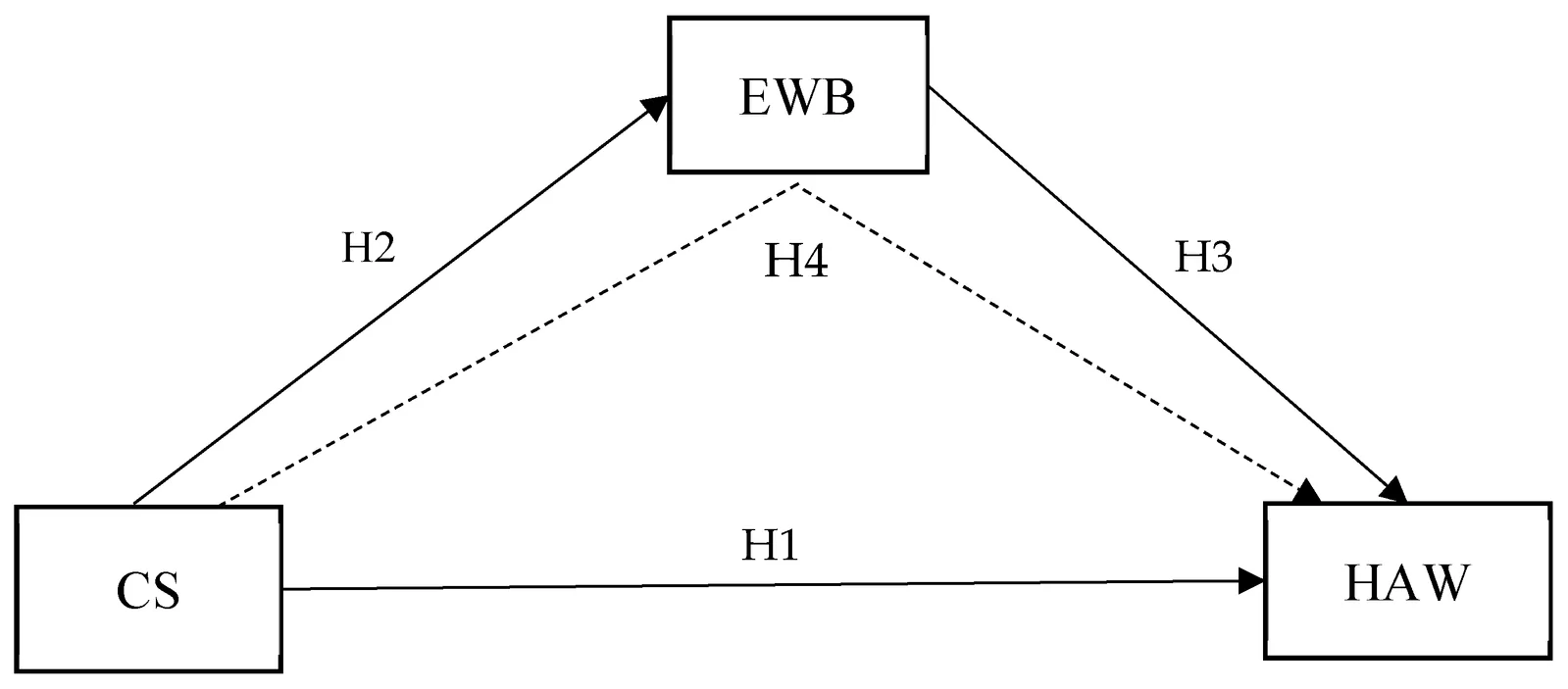
Research Model.

This study aimed to examine the relationship between nurses’ CS and HAW and to assess the mediating role of EWB in this relationship. The study used a cross-sectional correlational design. Figure 1 presents the study design.

### 2.2. Sampling and Study Participants

The study population consisted of nurses working at a tertiary care hospital in Sakarya Province, Türkiye. The required sample size was determined using G*Power software (version 3.1.9.7). An a priori power analysis based on linear multiple regression was conducted using the following parameters: small effect size (f^2^ = 0.05), alpha (α = 0.05), statistical power (1 − β = 0.95), and two predictors. Based on this analysis, the minimum total sample size required to test the study’s mediation hypotheses was calculated as 312.

Using convenience sampling, 500 questionnaires were distributed to nurses working in the hospital’s wards and intensive care units. A total of 416 questionnaires were returned, yielding a response rate of 83.2%. Of these, 21 were excluded because of incomplete responses or the presence of outliers. Data from the remaining 395 questionnaires were included in the analysis, and all statistical analyses were based on this sample. Thus, the final sample exceeded the calculated minimum sample size and met the targeted statistical power of 95%.

Table 1 presents the participants’ sociodemographic characteristics. Of the participants, 90.9% were female, 52.2% were single, 55.4% were aged 26–30 years, 82.0% held a bachelor’s degree, 63.3% had 1–5 years of experience, and 56.7% worked on inpatient wards.

### 2.3. Inclusion and/or Exclusion Criteria

The inclusion criteria were as follows: (1) providing direct patient care, (2) being a registered nurse working in an inpatient ward or intensive care unit, and (3) being available during the data collection period. The exclusion criteria were as follows: (1) completing a nursing internship, (2) working in an outpatient clinic, because clinical responsibilities in outpatient settings differ from those in inpatient units, and (3) declining to participate in the study.

### 2.4. Data Collection

Data were collected using a questionnaire. Participants were informed about the study’s purpose, and nurses who agreed to participate completed the questionnaire. Data collection took place between March and May 2025 during the nurses’ breaks, when they were not actively working. Each questionnaire took approximately 10–15 min to complete.

### 2.5. Data Collection Instruments

A two-part questionnaire was developed for this study. The first section consisted of a demographic information form developed based on a review of the relevant literature. It included items on participants’ age, gender, marital status, and educational background. The second section contained the CS, HAW, and EWB scales.

Compassion Satisfaction Scale: This unidimensional, 12-item scale was developed and validated by Nas and Sak (2021). The scale’s Cronbach’s alpha was reported as 0.92. Scores range from 12 to 60. Higher scores indicate higher levels of CS. The scale contains no reverse-scored items.

Happiness at Work Scale: The scale was developed by Salas-Vallina and Alegre (2018), and the validity and reliability of its Turkish version were examined by Bilginoğlu and Yozgat (2020). The scale’s Cronbach’s alpha was reported as 0.89. The scale has three subscales: work engagement, job satisfaction, and organizational commitment. The scale consists of nine items and contains no reverse-scored items.

Employee Well-being Scale: The scale was developed by Pradhan and Hati (2022), and the validity and reliability of its Turkish version were examined by Yüksel and Yılık (2022). The scale’s Cronbach’s alpha was reported as 0.912. The Turkish adaptation of the Employee Well-being Scale consists of four dimensions and 25 items. The four dimensions are social, psychological, workplace, and subjective well-being. Items are rated on a 5-point Likert scale, and two items are reverse-scored. The total score ranges from 25 to 125. Higher total and dimension scores indicate greater employee well-being, whereas lower scores indicate lower employee well-being.

### 2.6. Data Analysis

IBM SPSS Statistics 22.0 and the Hayes PROCESS macro (version 3.5) were used to analyze the study data. Frequencies and percentages were used to summarize the sociodemographic data; Cronbach’s alpha coefficient was used to assess internal consistency reliability; model fit indices generated using IBM SPSS Amos 22.0 were used to assess validity; correlation analysis was used to examine relationships among the study variables; and PROCESS Model 4 was used to test the hypotheses. In addition, the heterotrait–monotrait ratio of correlations (HTMT) was calculated to assess discriminant validity using the R programming language R 4.6.1 (R Core Team, 2026) and the semTools package 0.5-8 (Jorgensen et al., 2026).

### 2.7. Reliability and Validity Assessment

Cronbach’s alpha coefficients were calculated to assess the internal consistency reliability of the scales used in the study. The coefficients were 0.922 for the CS scale, 0.871 for the EWB scale, and 0.889 for the HAW scale. These values indicated that all three scales were reliable. The reliability analysis results are presented in Table 2.

Confirmatory factor analysis (CFA) was used to test the construct validity of the scales employed in the study (Erden et al., 2025). The results showed that the goodness-of-fit indices for the scales, including χ^2^/df, GFI, AGFI, CFI, NFI, TLI, and RMSEA, met the criteria for acceptable or excellent fit. For the EWB scale, the CFI indicated excellent fit, whereas the other indices indicated acceptable fit. For the HAW scale, the GFI, CFI, NFI, and TLI indicated excellent fit, whereas the other indices indicated acceptable fit. For the CS scale, the CFI indicated excellent fit, whereas the other indices indicated acceptable fit. Because the initial EWB scale model did not meet the criteria for convergent validity, items SWB6, SWB7, SWB8, PWB1, and SUWB1, which had factor loadings below 0.50, were excluded from the analysis. Table 3 presents the CFA results.

Convergent validity was also assessed using the average variance extracted (AVE) and composite reliability (CR) values proposed by Fornell and Larcker (1981). Table 4 presents the AVE and CR values for each scale. For convergent validity, AVE values of at least 0.50 and CR values of at least 0.70 are recommended (Ab Hamid et al., 2017; Hair et al., 2017). As shown in Table 4, all values met these thresholds, supporting the convergent validity of the measurement model.

The heterotrait–monotrait ratio of correlations (HTMT) was used to assess discriminant validity (Henseler et al., 2015). The HTMT values were calculated in R 4.6.1 (R Core Team, 2026) using the semTools package 0.5-8 (Jorgensen et al., 2026) and are presented in Table 5. All values were below the recommended threshold of 0.85 and ranged from 0.280 to 0.656, supporting discriminant validity among the study constructs.

## 3. Results

Table 6 presents the correlations among CS, EWB, and HAW. CS was significantly and positively correlated with both EWB (r = 0.531, *p* < 0.01) and HAW (r = 0.314, *p* < 0.01). EWB was also significantly and positively correlated with HAW (r = 0.638, *p* < 0.01).

Table 7 presents the results regarding the effect of nurses’ CS on HAW. The overall model was statistically significant (F = 43.052, *p* < 0.001). Nurses’ CS had a statistically significant positive total effect on HAW (β = 0.495, *p* < 0.001), which was subsequently decomposed into direct and indirect effects in the mediation analysis. Accordingly, H1 was supported.

Table 8 presents the findings on the effect of nurses’ CS on EWB. The model was statistically significant (F = 154.294; *p* < 0.001). According to the findings, CS had a statistically significant positive effect on EWB (β = 0.507; *p* < 0.001). The findings supported Hypothesis H2.

Table 9 presents the results regarding the effects of nurses’ CS and EWB on HAW. The overall model was statistically significant (F = 135.103, *p* < 0.001). After EWB was included in the model, the effect of CS on HAW was no longer statistically significant (B = −0.054, *p* > 0.05), whereas EWB had a statistically significant positive effect on HAW (B = 1.083, *p* < 0.001). The VIF was 1.393 for both predictors, indicating no evidence of multicollinearity. These results supported H3.

Table 10 presents findings on the mediating role of EWB in the relationship between nurses’ CS and HAW. According to the model, when employee well-being was included, the direct effect of CS on HAW was not statistically significant (β = −0.054; *p* > 0.05). The indirect effect was statistically significant (LLCI = 0.438; ULCI = 0.666). This indicates that employee well-being fully mediates the model. Based on these findings, Hypothesis H4 was supported.

## 4. Discussion

This study examined the relationship between nurses’ CS and HAW and the mediating role of EWB. The results showed that higher CS was associated with higher HAW and EWB among nurses. Similarly, in a study of nurses, Saliya et al. (2024) reported a positive relationship between CS and job satisfaction. Nadarajan et al. (2025) suggested that identifying the factors contributing to nurses’ CS may help improve HAW. Pradhan et al. (2024) found that employees who showed compassion toward others had higher levels of HAW. The present findings are therefore consistent with the existing literature. Therefore, greater CS may support nurses’ HAW.

The study also showed that CS was positively associated with EWB among the participating nurses. Durkin et al. (2016) and Zhang et al. (2025) reported that higher CS among nurses was associated with higher EWB. Yeşil and Polat (2023) suggested that psychoeducational interventions designed to enhance nurses’ compassion satisfaction may support their overall well-being. Among palliative care professionals, Galiana et al. (2022) also found a positive association between CS and EWB. From the perspective of self-determination theory, nurses may satisfy their need for competence through the fulfillment derived from compassionate care and their need for relatedness through the bonds they form with patients (Deci & Ryan, 2000). The present findings are therefore consistent with the existing literature.

The study found that EWB was positively associated with HAW. Previous research has also indicated a positive relationship between EWB and HAW. Mendoza-Ocasal et al. (2022) suggested that HAW may be fostered by improving the quality of work. Satisfaction with these psychological needs may contribute to nurses’ overall well-being. Van den Broeck et al. (2016) state that a sense of psychological fulfilment in the workplace is a factor that enhances employees’ general well-being. Faridi et al. (2025) reported that greater work experience was significantly associated with higher overall EWB and HAW among nurses. The field of positive organizational behavior has emerged from the theory of positive psychology, and one positive reflection of employee well-being is happiness (Xanthopoulou et al., 2012). The present results indicated full statistical mediation: when EWB was included in the model, the direct relationship between CS and HAW was no longer significant. This pattern can be interpreted through a central premise of SDT, according to which satisfaction of the basic psychological needs for autonomy, competence, and relatedness supports overall well-being (Ryan & Deci, 2000). Rayani et al. (2024) reported that providing care cultivates a sense of compassion among nurses and that this compassion contributes to their happiness and helps them feel more energetic at work. This state of well-being may then be reflected in subjective experiences across different areas of life. Orkibi and Ronen (2017) similarly concluded that individual competence did not directly increase subjective well-being but had an indirect effect through the satisfaction of psychological needs. The present results suggest that CS is related to HAW through higher EWB. They also indicate that nurses’ well-being and workplace happiness are closely connected. Nurses with higher levels of well-being may therefore report greater workplace happiness because they carry this positive psychological state into their workplace experiences.

### Limitation and Future Direction

The correlational, cross-sectional design limits causal inferences regarding the relationships among the study variables (Taris & Kompier, 2006). In addition, sociodemographic characteristics were not included as covariates in the mediation analysis to maintain a simple model. While the findings show statistical associations and indirect effects consistent with the proposed theoretical model, the temporal ordering of the variables cannot be determined. Therefore, alternative explanations and reverse relationships cannot be ruled out. Future longitudinal or experimental studies may provide stronger evidence about the temporal ordering of these relationships and their potential causal mechanisms. However, the current findings provide useful preliminary evidence regarding the relationships among the study variables and may contribute to the further development of the theoretical framework in this field. Because the data were collected from nurses at a single hospital using convenience sampling, the sample may not fully represent the broader population of nurses. Collecting data at a single hospital limits the generalizability of the findings to other hospitals and healthcare systems. Because organizational culture, management practices, working conditions, and employee experiences may differ across hospitals, the findings should be interpreted with caution.

## 5. Conclusions

The findings indicate that EWB statistically mediates the relationship between nurses’ CS and HAW, with full mediation observed in the tested model. This result shows the importance of supporting nurses’ EWB. After EWB was included in the model, the direct relationship between CS and HAW was no longer significant, whereas higher EWB was associated with higher HAW. Nurses may experience CS when providing patients with the care and treatment they need, and this satisfaction may be indirectly related to their HAW through EWB. Sustaining higher HAW may therefore depend, in part, on strengthening nurses’ EWB. In conclusion, the findings suggest that prioritizing employee well-being in nursing management may benefit both nurses and healthcare organizations.

Because compassion satisfaction is positively associated with happiness at work, nurses’ dedicated caregiving efforts should be supported and encouraged through recognition and rewards. In this context, nurses could be formally recognized with plaques at institutional events. In addition, given that EWB fully mediated the relationship between CS and HAW in the tested model, workplace practices that support EWB could be developed through feedback mechanisms that regularly assess nurses’ needs, expectations, and experiences. In this context, staffing levels could be adjusted to ensure adequate nurse coverage in inpatient wards and intensive care units, a nurse-supportive leadership approach could be adopted, and flexible work arrangements could be implemented. These measures may enhance nurses’ happiness at work, which may, in turn, improve the quality of care. In addition, future studies could incorporate theoretically relevant individual-difference variables, such as personality traits, psychological capital, or resilience, into the proposed model. Including these variables may provide a more comprehensive understanding of how these relationships operate.

## Figures and Tables

**Table 1:** Socio-demographic Characteristics.

Variable	Category	*n*	%
Gender	Male	36	9.1
	Female	359	90.9
Marital Status	Married	189	47.8
	Single	206	52.2
Age	≤25	68	17.2
	26–30	219	55.4
	31–35	70	17.7
	≥36	38	9.6
Educational Level	High School	16	4.1
	Associate Degree	26	6.6
	Bachelor’s Degree	324	82.0
	Graduate Degree	29	7.3
Professional Experience (Year)	≤5	250	63.3
	6–10	83	21.0
	11–15	31	7.8
	≥16	31	7.8
Working Unit	Inpatient Ward	224	56.7
	Intensive Care Unit	171	43.3
Total		395	100

**Table 2:** Cronbach Alpha Values.

Scale	Cronbach Alpha	Number of Items
CS	0.922	12
EWB	0.871	20
HAW	0.889	9

**Table 3:** Model Fit Summary.

Assessment Index for Model Fit	Normal Value	Allowable Value	Value of Index EWB	Value of Index HAW	Value of Index CS
CMIN/DF (χ^2^/df)	<2	<5	2.006	3.282	3.129
GFI	>0.95	>0.90	0.925	0.960	0.932
AGFI	>0.95	>0.85	0.902	0.924	0.897
CFI	>0.95	>0.90	0.951	0.975	0.958
NFI	>0.95	>0.90	0.907	0.965	0.940
TLI	>0.95	>0.90	0.942	0.963	0.946
RMSEA	<0.05	<0.08	0.051	0.076	0.074

Source: (Erden et al., 2025).

**Table 4:** Discriminant Validity Results (Fornell–Larcker Criterion).

Scale	AVE	$\sqrt{AVE}$	CR
CS	0.507	0.712	0.924
EWB	0.501	0.708	0.951
HAW	0.656	0.810	0.944

**Table 5:** HTMT Values for Discriminant Validity.

Scale	CS	EWB	HAW
CS	1		
EWB	0.648	1	
HAW	0.280	0.656	1

**Table 6:** The Relationship Between Compassion Satisfaction, Employee Well-being, and Happiness at Work.

	1	2	3
CS (1)	1		
EWB (2)	0.531 **	1	
HAW (3)	0.314 **	0.638 **	1

** Correlation is significant at the 0.01 level (2-tailed).

**Table 7:** The Effect of Compassion Satisfaction on Happiness at Work.

Dependent Variable	Independent Variable	Regression						Model Summary		
		β	SE	t	*p*	LLCI	ULCI	R^2^	F	*p*
HAW	Constant	0.689	0.322	2.137	0.033	0.055	1.323	0.099	43.052	0.000
	CS	0.495	0.075	6.561	0.000	0.347	0.644			

Note: SE = Standard Error; LLCI = Lower Limit Confidence Interval; ULCI = Upper Limit Confidence Interval.

**Table 8:** The Effect of Compassion Satisfaction on Employee Well-being.

Dependent Variable	Independent Variable	Regression						Model Summary		
		β	SE	t	*p*	LLCI	ULCI	R^2^	F	*p*
EWB	Constant	1.619	0.174	9.283	0.000	1.276	1.962	0.282	154.294	0.000
	CS	0.507	0.041	12.422	0.000	0.427	0.587			

Note: SE = Standard Error; LLCI = Lower Limit Confidence Interval; ULCI = Upper Limit Confidence Interval.

**Table 9:** The Effect of Compassion Satisfaction and Employee Well-being on Happiness at Work.

Dependent Variable	Independent Variable	Regression							Model Summary		
		β	SE	t	*p*	LLCI	ULCI	VIF	R^2^	F	*p*
HAW	Constant	−1.064	0.289	−3.684	0.000	−1.632	−0.496		0.408	135.103	0.000
	CS	−0.054	0.072	−0.747	0.455	−0.196	0.088	1.393			
	EWB	1.083	0.076	14.312	0.000	0.934	1.232	1.393			

Note: SE = Standard Error; LLCI = Lower Limit Confidence Interval; ULCI = Upper Limit Confidence Interval.

**Table 10:** The Mediating Role of Employee Well-being in the Effect of Compassion Satisfaction on Happiness at Work.

Direct Effect			Effect	SE	t	*p*	LLCI	ULCI
CS		HAW	−0.054	0.072	−0.747	0.455	−0.196	0.088
Indirect Effect								
CS	EWB	HAW	0.549	0.058			0.438	0.666
Total Effect			0.495	0.075	6.561	0.000	0.347	0.644

Note: SE = Standard Error; LLCI = Lower Limit Confidence Interval; ULCI = Upper Limit Confidence Interval.

## Data Availability

The datasets used and analyzed in this research are available from the corresponding author upon reasonable request.

## Abbreviations

The following abbreviations are used in this manuscript:

AGFI	Adjusted Goodness of Fit Index
CFI	Comparative Fit Index
CMIN/DF	Chi-square/Degrees of Freedom
GFI	Goodness of Fit Index
LLCI	Lower-Level Confidence Interval
NFI	Normed Fit Index
RMSEA	Root Mean Square Error of Approximation
S.E.	Standard Error
SDT	Self-Determination Theory
TLI	Tucker–Lewis Index
ULCI	Upper-Level Confidence Interval
